# Potential Response to Selection of HSP70 as a Component of Innate Immunity in the Abalone *Haliotis rufescens*

**DOI:** 10.1371/journal.pone.0141959

**Published:** 2015-11-03

**Authors:** Katherina B. Brokordt, Roxana C. González, William J. Farías, Federico M. Winkler

**Affiliations:** 1 Centro de Estudios Avanzados en Zonas Áridas (CEAZA), Coquimbo, Chile; 2 Departamento de Biología Marina, Facultad de Ciencias del Mar, Universidad Católica del Norte, Coquimbo, Chile; Fred Hutchinson Cancer Research Center, UNITED STATES

## Abstract

Assessing components of the immune system may reflect disease resistance. In some invertebrates, heat shock proteins (HSPs) are immune effectors and have been described as potent activators of the innate immune response. Several diseases have become a threat to abalone farming worldwide; therefore, increasing disease resistance is considered to be a long-term goal for breeding programs. A trait will respond to selection only if it is determined partially by additive genetic variation. The aim of this study was to estimate the heritability (*h* ^2^) and the additive genetic coefficient of variation (*CV*_A_) of HSP70 as a component of innate immunity of the abalone *Haliotis rufescens*, in order to assess its potential response to selection. These genetic components were estimated for the variations in the intracellular (in haemocytes) and extracellular (serum) protein levels of HSP70 in response to an immunostimulant agent in 60 full-sib families of *H*. *rufescens*. Levels of HSP70 were measured twice in the same individuals, first when they were young and again when they were pre-harvest adults, to estimate the repeatability (*R*), the *h* ^2^ and the potential response to selection of these traits at these life stages. High HSP70 levels were observed in abalones subjected to immunostimulation in both the intracellular and extracellular haemolymph fractions. This is the first time that changes in serum levels of HSP70 have been reported in response to an immune challenge in molluscs. HSP70 levels in both fractions and at both ages showed low *h* ^2^ and *R*, with values that were not significantly different from zero. However, HSP70 induced levels had a *CV*_A_ of 13.3–16.2% in young adults and of 2.7–8.1% in pre-harvest adults. Thus, despite its low *h* ^2^, HSP70 synthesis in response to an immune challenge in red abalone has the potential to evolve through selection because of its large phenotypic variation and the presence of additive genetic variance, especially in young animals.

## Introduction

Disease resistance may be reflective of immune function and can be measured by assessing the resistance of the organism to a particular pathogen or by measuring components of the immune system [1, 2] Similar to the majority of invertebrates, abalone only possess an innate immune system with humoral and cellular (mediated by haemocytes) defence mechanisms [3]. Haemocytes recognise the pathogen-associated molecular patterns (PAMPs) in microorganisms and mount an immune response to eliminate foreign organisms directly by scavenging (cellular response) or by releasing molecules that destroy the microorganisms or mitigate damage they caused (humoral response). HSPs (heat shock proteins), which have the primary roles as molecular chaperones and to fold/refold proteins, also are among the molecules synthesised as part of the humoral response [4]. HSPs are immune effectors (acute-phase proteins) and have been described as potent activators of the innate immune system in some invertebrates [3]. HSP70 is the most studied family of HSPs, and increases in HSP70 levels have been observed in several marine invertebrates, such as gastropods, bivalves, crustaceans and corals in response to various pathogens [5–13]. It goes without saying that the immunomodulatory function of HSP70 has been well studied in vertebrates [14–16]; it is known to have both intracellular (cytoprotective/anti-apoptotic) and extracellular functions (immunogenic) [4]. However, these immune functions have been less studied in invertebrates, although HSP70 appears to have similar roles to those described for vertebrates [17].

Aquaculture production systems favour the transmission and spread of disease because of high animal densities and stressful changing environments (e.g. temperature, oxygen, ammonia) [18]. Disease outbreaks cause important losses in aquaculture production [19], therefore, increasing disease resistance is considered to be a long-term goal for breeding programs [20].

The farming of abalone has undergone robust worldwide growth mainly because of their high commercial value and the decline of natural stocks due to overexploitation [21]. In Chile, abalone production (mainly the California red abalone, *Haliotis rufescens*) has increased markedly in the last decade [22]. However, several diseases have become a threat to abalone farming in Chile and worldwide [23–27].

Because molluscs do not produce antibodies, vaccines cannot be used to protect them against infectious diseases. Furthermore, the use of preventive drug therapies is usually restricted because much shellfish culture occurs in the natural marine environment [28]. The strategy to improve the animals' resistance using selective breeding programs has the advantage that breeding is directional, low cost and easy to implement [20]. However, the capability of a quantitative trait to respond to selection depends on the existence of genetic variation (*V*_G_) for the trait in the population. The *V*_G_ can be split into an additive component (*V*_A_) and non additive genetic components (dominance and interaction), being *V*_A_ the responsible of the resemblance between relatives [29]. Narrow-sense heritability (*h* ^2^) is the proportion of the total phenotypic variance (*V*_P_) caused by additive genetic variance (*h* ^2^ = *V*_A_/*V*_P_) [29]. Therefore, a low *h* ^2^ may arise from a low *V*_A_, a high environmental variance, or a combination. The magnitude of intergenerational response to directional selection on the phenotype is proportional to *h* ^2^ [29]. An alternative measure of a trait′s potential to respond to selection is the additive genetic coefficient of variation (*CV*_A_). This estimate of evolvability standardizes the estimates of *V*_A_ with respect to the mean value of the trait [30–32]. Several studies in mammals [33], insects [34, 35] and fish [36], and one study in molluscs [37] have shown that disease resistance traits often have moderate to high *h* ^2^; however, the results are specific to the particular pathogen being studied and other non-immune factors, such as epistatic genotype-genotype interactions, may confound the results [38]. An alternative is to quantify the genetic variability of immune function (immunocompetence), which provides an indication of nonspecific immune response instead of the capacity to resist a specific pathogen [38, 39]. Quantitative genetic studies of the immune response in various invertebrates have shown that *h* ^2^ can vary from moderate (*h* ^2^ = 0.20–0.36, for haemocyte density) to high (*h* ^2^ = 0.83 and 0.65 for phagocytic capacity and phenoloxidase activity, respectively) for these immune-response traits [2, 38, 40, 41].

The aim of this study was to estimate the potential response to selection, by estimating *CV*_A_ and *h* ^*2*^, of the variation of intracellular and extracellular HSP70 protein levels in response to an immunostimulant agent in two life stages, young adults and pre-harvest adults, of the red abalone, *Haliotis rufescens*. The genetic and environmental factors affecting trait variation may change during ontogeny, making the future performance of individuals difficult to predict [42]. Therefore, the availability of genetic parameters such as *h* ^2^ and *CV*_A_ of the studied traits at different life stages, as well as the consistency of the between individuals variation for these traits, i.e., their repeatability (*R*), is essential for making decisions concerning the design and implementation of selective breeding programs [43]. From an evolutionary point of view, *h* ^2^ and *CV*_A_ of the studied innate immune traits in the red abalone can reveal whether these traits can evolve through natural selection.

## Materials and Methods

### Breeding design and animal rearing conditions

Sixty full-sib families were produced using *Haliotis rufescens* abalone broodstock that was randomly obtained from a base population of 600 adults, provided by three abalone breeding companies (200 abalone per company). The broodstock were conditioned over four months in 2000-L tanks filled with micro-filtered seawater at a temperature between 18°C and 19°C. Permanent feeding with macroalgae was provided. The mature broodstock were induced to spawn separately using the hydrogen peroxide method [44], and crossing was conducted following a paternal half-sib nested design. Seawater containing gametes of one male was used to fertilise oocytes from three females randomly chosen from the base population, and a total of 20 males and 60 females were used. After fertilisation, the zygotes from each family were transposed into individual 20-L polycarbonate containers containing filtered and UV-treated sea water, washed several times with filtered sea water to eliminate excess spermatozoa, and retained on a 60-μm mesh net. After hatching, the larvae from each full-sib family were allowed to grow for 5–6 days with gentle aeration and daily water changes. Subsequently, the competent larvae were transferred to 200-L tanks provided with corrugated polycarbonate plates inoculated with wild benthic microalgae for settling.

The production of the families occurred over ~3 months, with three spawning events per month (5–10 days between each event). After settling, each full-sib family was cultured separately in 200-L tanks with continuous water flow and constant aeration for the first 14 months. Young were initially fed with wild benthic microalgae inoculated on corrugated polycarbonate plates. From the seventh month onward, the abalones were fed fresh kelp (*Macrocystis pyrifera*). Upon reaching a shell length of ≥ 20 mm (~14 months), the abalone had individual labels attached to the shell with epoxy resin. Subsequently, individuals from different families were mixed and transferred to baskets placed in a 10,000-L raceway-type tank. The abalone were maintained for three years in the raceway (i.e., until they attained an age of four years) with continuous water flow and constant aeration at ambient temperature that varied between ~13 and 20°C and were fed twice a week with *M*. *pyrifera*.

HSP70 induction was measured twice. The first measurement was performed when the abalone were three years old (young adult ∼40 mm shell length), and the second measurement was performed when they were four years old (adults ∼60 mm). For this work, between 5 and 10 individuals per family of full siblings were evaluated (n = 492 and 357 per year, respectively).

### Immune challenge and haemolymph collection

Preliminary experiments were conducted to determine the type of agent (immunostimulant) that most reliably induced an increase of HSP70 in the haemocyte and serum. We evaluated two β-glucans (laminarin and zymosan) and a bacterium (*Vibrio splendidus*), injected in different doses (60 and 100 μg per individual for β-glucans; and 8×10^5^, 1×10^6^ and 1×10^8^ CFU per individual for the bacterium); and also evaluated several post-challenge times (6, 12, 24 and 48 h post-challenge). For each of these assays, we used individuals injected with the same volume of sterilised seawater (SSW) and uninjected abalones, as controls. These preliminary experiments indicated that β-glucan zymosan injected in the higher dose (100 μg per individual) induced the highest HPS70 levels. The largest difference between the controls and challenged individuals was observed at 24 h. The advantage of this immunostimulant compared with bacteria is that zymosan can be applied in exact and equivalent doses to each individual. Such precision is key in determining the genetic component of phenotypic variance.

Based on these results, the immune challenge of the abalone belonging to the 60 full-sib families used the following protocol: 100 μl of a solution of 0.1 mg μl^-1^ zymosan (Sigma-Aldrich, St. Louis, MO, USA) diluted in SSW was injected in the foot of each abalone with a 25 G 5/8” syringe. Subsequently, the animals were returned to the experimental tank. After 24 h, 400 μl of haemolymph was sampled from the foot of each abalone. The haemolymph was then centrifuged at 600 x *g* over 5 min to separate the haemocytes (cellular fraction) from the serum (extracellular fraction). The haemocytes and serum were maintained in 200 μl of preservation buffer (32 mM Tris-HCl pH 7.5, 1 mM ethylenediaminetetraacetic acid (EDTA), 1 mM Pefabloc, 1 mM protease inhibition cocktail), frozen in liquid nitrogen and stored at -80°C for subsequent analysis of HSP70.

### Extraction and quantification of total protein

To extract the proteins from the haemocytes, we froze (at -80°C) and thawed the samples (2 cycles), then mixed them for 5 min, by vortex, and incubated them at 95°C for 5 min. The homogenate was centrifuged at 10,600 x *g* for 20 min, and the supernatant was removed and stored at -80°C. To extract proteins from the serum, the sample was mixed by vortexing for 5 min. The homogenate was centrifuged at 10,600 x *g* for 20 min, and the supernatant was removed and stored at -80°C. Total protein was quantified in an aliquot of the supernatants using a Micro-BCA kit (Novagen, San Diego, CA, USA) using a microplate spectrophotometer EPOCH (BioTek, Winooski, VT, USA).

### HSP70 quantification

HSP70 from the haemocytes and serum of each abalone were measured by enzyme-linked immunosorbent assay (ELISA) that was previously validated by performing Western-blot analyses with the primary antibody [polyclonal mono-specific anti-epitope for HSP70, developed in immunized mice with a synthetic peptide epitope (Group of Immunological Markers on Aquatic Organisms, Catholic University of Valparaiso, Chile)]. To determine whether the primary antibody would recognize an inducible HSP70 isoform, quantifications were performed on the haemocytes and serum of challenged, positive-control, and negative-control abalone. Challenged abalone were injected with a solution of zymosan and sterile sea water (SSW); positive-control abalone were exposed to heat shock (i.e., an increase of 8°C during one hour); and negative-control abalone were not injected nor exposed to heat shock. For abalone that were challenged and exposed to heat shock, only one band at the level of 70 kD-HSP was observed, which is indicative of the detection of one HSP70 isoform; whereas, no band was detected for negative control abalones, suggesting that the primary antibody recognized an inducible HSP70 isoform (S1 Fig).

Total protein (30 μg/mL) was diluted in 0.05 M carbonate-bicarbonate buffer (pH 9.6), and 50 μL of the sample was aliquoted per well and incubated in an ELISA plate overnight at 4°C with three blanks (buffer only) and various concentrations of cognate HSP70 (to generate a standard curve). The plate was then washed twice with phosphate-buffered saline (PBS) (200 μL per well), and 200 μL of blocking buffer (PBS + 5% skim milk) was subsequently added to each well and incubated for 2 h. The wells were washed again with PBS. Subsequently, 100 μL (40 ng/μL) of the primary antibody diluted 1:400 in blocking buffer + 0.05% Tween-20 was added to each well and incubated overnight at 4°C. The plate was then washed four times with PBS, incubated with a goat anti-mouse IgG secondary polyclonal antibody (Thermo Fisher Scientific Cat# PA1-31432, RRID:AB_2158025), diluted at 1:2500 in blocking buffer + 0.05% Tween-20 for 2 h at 25°C, and washed again four times with PBS. Then, 100 μL of substrate solution (10 mg *o*-phenylenediamine dihydrochloride in 25 mL of 0.05 M citrate phosphate buffer) was added, and the plate was incubated for 30 min at 25°C. Finally, the plate was read at 450 nm in a microplate spectrophotometer. The absorbance of the sample was corrected by the mean absorbance of the blanks and divided by a conversion factor, which was estimated from a linear regression curve of cognate HSP70. The calculated result was the concentration of HSP70 in μg/mg total protein.

The effect of immune challenge with zymosan on HSP70 induction was assessed by one-way analysis of variance (ANOVA) for the cell and serum fractions. A comparison of the HSP70 levels according to the year of measurement (life stages) and haemolymph fraction for the entire population was performed by two-way ANOVA. For both ANOVAs, normality of the dependent variable was tested using the Shapiro-Wilks test [45] and homogeneity of variances using the Levene test [46] to verify that the data met model assumptions. *A posteriori* tests for specific differences were conducted via multiple pairwise comparisons of Least-Square Means [47], with significance evaluated at *P* ≤ 0.05. We applied a sequential Bonferroni procedure to correct for type I error in multiple simultaneous tests [45].

### Estimation of the components of phenotypic variance and heritability

The heritability and variance components for the intracellular and extracellular HSP70 levels were estimated with a restricted, estimated maximum-likelihood (REML) procedure [48] as implemented in ASReml v.3.0 [49]. This procedure involved fitting an individual animal model, i.e, a mixed linear model where the phenotypic response of each individual is separated into an additive genetic component plus other random and fixed effects, as follows (in matrix notation):

$$y=Xb+Z_{a}a+Z_{m}f+e$$

where ***y*** is a vector of the observations of all individuals; **b** is the vector of fixed effects; **a** is the vector of additive genetic effects (random animal effects or breeding values); **f** is the vector of random effects other than additive genetics (i.e., confounded maternal effects, common environmental effects as well as non-additive genetic effects); and ***e*** represents the residual effects. ***X***, ***Z***_***a***_ *and* ***Z***_***m***_ are the corresponding incidence matrices.

Using this model, we estimated the significance of the fixed effects and covariates using the Wald F statistic. The statistical significance of the maternal/environmental/non-additive random effects and additive random effects (*h* ^2^ significance) was estimated by the log-likelihood ratio test (log-LR test). The variables that were evaluated as fixed effects were the 1) location of the tank in which each full-sib family was held for the first 14 months of life, 2) densities at which the families were held during this period, 3) order in which haemolymph was sampled and 4) year of the measurements (i.e., year 1 or 2). We also evaluated the effects of the exact age at the time of the measurements within a year as a covariate. As random factors in the model, we evaluated the direct additive genetic effects, as well as the maternal/common environmental effects. Because full siblings shared a tank for 14 months, the early common environmental effects were completely confounded with the maternal effects. In addition, potential non-additive genetic effects are also confounded with maternal effects and cannot be teased out with the experimental design followed here. The direct *h* ^2^ of each trait was calculated as the ratio of the additive genetic variance to the total phenotypic variance [29, 31, 50]. The *h* ^2^ of HSP70-induced expression was estimated for each year class (young adults and near-harvest adults) separately and combined.

The repeatability (*R*) of the HSP70 level in the haemocytes and serum was estimated through ASReml v.3.0 [49] using the responses for the two abalone year classes together. *R* estimates the consistency of the among-individuals variation for a trait; in other words, if an individual’s relative ranking in the population remains constant over time [51].

For the comparison of the additive genetic and residual variance across different HSP70 expression traits, we calculated coefficients of additive genetic variance (*CV*_A_ = 100√*V*_A_/*X*) and residual variance (*CV*_R_ = 100√*V*_P_—*V*_A_/*X*). This calculation includes trait means (*X*), which are considered to be more appropriate for standardising the variances and inferring the evolvability of quantitative traits [30].

### Ethics statement

The abalones used in this study were provided by three private abalone breeding companies (Live Seafood Chile S.A., Abalones Chile S.A. and Cultivos Marinos San Cristobal S.A.). Abalone transport form each private company to the Universidad Católica del Norte Aquaculture Center, was permitted and supervised by the National Service of Fisheries of Chile. Ethical approval was not required for this study because no endangered animals were involved.

Animal maintenance and experimental manipulations in this study were carried out in strict accordance with the recommendations in the CCAC guidelines on: choosing an appropriate endpoint in experiments using animals for research, teaching and testing (http://www.ccac.ca/Documents/Standards/Guidelines). All efforts were made to minimize suffering during animal manipulations and surgery. The protocol for sampling procedures and experimental manipulations were reviewed and approved by the Bioethics Committee of the Centro de Estudios Avanzados en Zonas Aridas (Permit Number: 005–13) and the National Council of Science and Technology of Chile.

## Results

### Phenotypic responses

**Fig 1:**
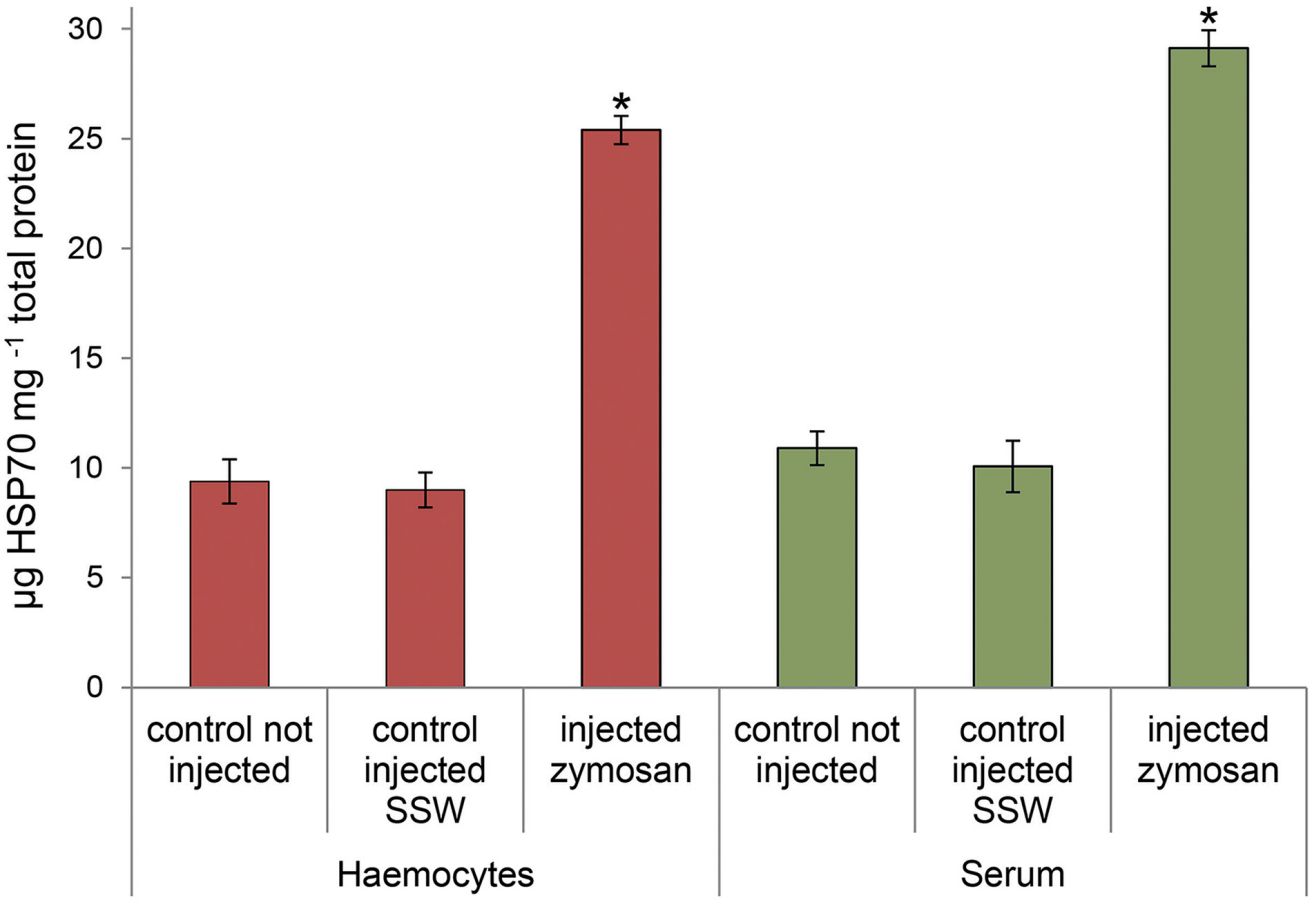
Induced levels of HSP70 in haemocytes and serum of challenged red abalone *Haliotis rufescens*. HSP70 levels were measured in the intracellular (haemocytes) and extracellular (serum) fractions of *H*. *rufescens*, in individual not injected (i.e., injection control), injected with sterilized sea water (SSW) (i.e., challenge control) and injected with the β-glucan, zymosan (24-h post-injection). The data are shown as X ± S.E., n = 6–10 per condition. (*) indicates significance (*P* < 0.01) between treatment and controls at the indicated haemolymph fraction.

Injection of the β-glucan, zymosan, into the abalone foot led to a marked induction of HSP70 levels both in the haemocytes (cellular fraction) and serum. After 24 h, individuals injected with zymosan had approximately 3-fold higher levels of HSP70 in the haemocytes and serum compared with those of non-injected control abalone or control abalone injected with sterilised seawater (SSW) (*P* < 0.01) (Fig 1).

Young red abalone adults exposed to the immunostimulant agent zymosan showed a lower average expression of HSP70 in the cellular (haemocytes) and extracellular (serum) fractions compared to near-harvest adult abalones (Table 1). In the case of haemocytes HSP70 expression, it was ~50% lower in the young adults than in near-harvest adults.

**Table 1:** Phenotypic means and genetic estimates for HSP70 induced levels, measured in the haemocyte (intracellular) and serum (extracellular) fractions of the haemolymph of challenged *Haliotis rufescens* at two development stages, young adults and near harvest adults.

Immunologic traits (μg HSP70/mg total protein)	Phenotypic Mean (SD)	*V*_A_ (SE)	*V*_R_ (SE)	*V*_P_ (SE)	*h* ^2^ (SE)	*CV*_A_ (%)	*CV*_R_ (%)
**Intracellular HSP70 young adults (S-m)** ^**a**^	16.40 (7.12) ^c^	4.72 (3.58)	45.91 (4.58)	50.62 (3.83)	0.09 (0.07)	13.25	41.32
**Intracellular HSP70 near harvest adults (SO-m)** ^**b**^	32.27 (10.25) ^a^	0.76 (3.98)	92.29 (7.16)	93.06 (6.11)	0.01 (0.04)	2.70	29.77
**Extracellular HSP70 young adults (S-m)**	26.59 (19.34) ^b^	18.61 (26.07)	355.85 (36.46)	374.47 (29.39)	0.05 (0.07)	16.22	70.94
**Extracellular HSP70 near harvest adults (SO-m)**	30.87 (11.50) ^a^	6.31 (5.11)	84.14 (6.98)	90.44 (5.94)	0.07 (0.06)	8.14	29.71

Genetic estimates of the additive genetic variance (*V*_A_); residual variance (*V*_R_); phenotypic variance (*V*_P_); heritability (*h* ^*2*^); and coefficients of additive genetic variance (*CV*_A_) and residual variance (*CV*_R_).

Immune response through HSP70 expression was assessed in abalone belonging to 60 full-sib families (n = 492 and 357 for young and near harvest adults, respectively). Genetic parameters were estimated using two models ^a^(S-m, simple model that only included the additive genetic effect as a random effect for young adults; and ^b^SO-m, model that also included the order of sampling as a fixed effect for near harvest adults). Models changed depending on the factors that were significant at a specific age. Phenotypic means were compared by two-way ANOVA (different letters denote significant differences at *P* < 0.01)

### Genetic estimates

Among the factors evaluated to define the linear mixed model for the separate analysis of the two age groups (young adults and pre-harvest adults), only the sampling order had a significant effect in the pre-harvest adults (Wald F, *P* < 0.001). No significant maternal/common environment effects were detected for either life stage (log-LR test, *P* > 0.05). No differences due to sampling order or other fixed factors were detected for young adults (Wald F, *P* = 0.228 and 0.59 for haemocytes and serum, respectively). Therefore, estimates of the variances and *h* ^2^ for the young adults were made through a reduced or simple model (S-m) that only included the additive genetic effect as a random effect; and for pre-harvest adults the estimates were made through a model that also included the order of sampling as a fixed effect (So-m) (Table 1). In the analysis of the linear mixed model that combined the two age classes, the sampling order nested in the year of sampling and age had significant effects (Wald F, *P* < 0.001). Therefore, the estimates of variance, *h* ^2^ and *R* considered these factors, and two models were generated. Model 1 used the sampling order nested in the year of sampling as fixed factor, and Model 2 used the age of the abalone at the time of measurement as co-variable (Table 2).

**Table 2:** Phenotypic means and genetic estimates for HSP70 induced levels, measured in the haemocyte (intracellular) and serum (extracellular) fractions of the haemolymph of challenged *Haliotis rufescens* considering the two developmental stages together.

	Phenotypic Mean (SD)	*V*_A_ (SE)	*V*_R_ (SE)	*V*_P_ (SE)	*R* (SE)	*h* ^2^ (SE)	*CV*_A_ (%)	*CV*_*R*_ (%)
**Model 1**								
**Intracellular HSP70**	25.39 (11.97)	7×10^−7^ (4×10^−8^)	75.07 (3.72)	75.07 (3.72)	NE	0.00 (0.00)	0.01	34.12
**Extracellular HSP70**	29.12 (15.34)	7.66 (6.61)	206.14 (11.77)	213.81 (10.78)	0.04 (0.03)	0.04 (0.03)	9.50	49.30
**Model 2**								
**Intracellular HSP70**	25.39 (11.97)	3.22 (2.46)	79.66 (4.44)	82.88 (4.11)	0.04 (0.03)	0.04 (0.03)	7.07	35.15
**Extracellular HSP70**	29.12 (15.34)	5.95 (6.74)	224.84 (12.71)	230.79 (11.58)	0.03 (0.03)	0.03 (0.03)	8.38	51.49

Genetic estimates of the additive genetic variance (*V*_A_); residual variance (*V*_R_); phenotypic variance (*V*_P_); heritability (*h* ^*2*^); and coefficients of additive genetic variance (*CV*_A_) and residual variance (*CV*_R_).

Immune response through HSP70 expression was measured two times in abalone belonging to 60 full-sib families (young and near harvest adults; n = 849 measures). Genetic parameters were estimated using two models. **Model 1** included the order of sampling nested in the year of sampling as a fixed effect; and **Model 2** included the age of the abalone at the moment of sampling as a co-variable.

The additive genetic variance (*V*_A_) of the intracellular (haemocytes) and extracellular (serum) HSP70 expression was low compared with the residual variance (*V*_R_) in both young adults and pre-harvest adults (Table 1). Accordingly, the estimated *h* ^2^ of HSP70 variation in both fractions was low (0.01–0.09) and not significantly different from zero (log-LR test, *P* > 0.05) for both fractions and at both ages. The coefficients of additive genetic variation (*CV*_A_) and residual variation (*CV*_R_) in the haemocytes and serum showed much higher values in young adults (13.25–16.22%) than in pre-harvest adults (2.7–8.14%) (Table 1).

The variances for the intracellular and extracellular HSP70 when the information from both of the age classes were combined and estimated from the two mixed models, showed high *V*_R_ and low *V*_A_ (Table 2) and resulted in low and not significant *h* ^2^ (log-LR test, *P* > 0.05) for HSP70 in the two haemolymph fractions. In addition, the *R* of HSP70 expression between the young adult and pre-harvest adult abalones was estimated with both models. Low *R* values (0–0.04) were detected for the expression of this protein in the haemocyte and serum (Table 2). The *CV*_A_ for intracellular HSP70 estimated with Model 1 was close to zero, whereas the remaining estimates for HSP70 in both fractions ranged from 7–9% using both Model 1 and 2 (Table 2). The *CV*_R_ estimated using both models was high for both fractions of the haemolymph.

## Discussion

The intracellular and extracellular induction of HSP70 in challenged young and near-harvest *H*. *rufescens* presented low *h* ^2^ that did not differ from zero. The high *V*_R_ of induced HSP70 levels suggests that the variation of synthesis under immunostimulation is greatly affected by environmental factors and/or non-additive genetic effects (dominance or genetic interaction).

The consistency between individual variation is usually estimated by *R* [52], which specifically estimates if an individual’s relative ranking in the population remains constant over time [51]. Because *R* includes all of the components of genetic variance in addition to the common environment variance (*R* = [*V*_Ec_+*V*_G_]/*V*_P_), it sets the approximate upper limit for *h* ^2^ [29, 53]. In the present study, the intracellular and extracellular induced levels of HSP70 measured in the same individuals at 3 and 4 years of age showed low *R* (0.03–0.04); thus these traits are not consistent over this time period. The magnitude of *R* was consistent with the estimated *h* ^2^ for the same traits, suggesting that non-additive genetic and common environment factors have a negligible effect on the induction of HSP70 synthesis in challenged abalone.

The immune response is the primary defence strategy for organisms against invading agents. This trait directly affects fitness (ability to leave fertile offspring) because it can influence survival or reproductive capacity [37]. In this sense our estimates of *h* ^*2*^ for HSP70 levels as an immune response are consistent with Fisher’s theorem (1930), which proposes that key traits for fitness under strong directional selection would have very low *h* ^2^ and observable variation should essentially be caused by non-genetic additive causes [54]. However, the low *h* ^2^ of a trait may be the result of high residual variance and not low additive genetic variability [30, 55]. The *CV*_A_ indicates the ability of a given trait to respond to selection (“evolvability”) and facilitates inference of the forces that maintain genetic variability [30]. Because *h* ^*2*^ = *V*_A_/(*V*_R_+*V*_A_), the *CV*_A_ and *CV*_R_, can provide information on the possible causes of low *h* ^*2*^ values in fitness-related traits [32]. In this study, the *CV*_A_ varied between 2.7 and 16.2% for induced levels of HSP70. These results are consistent with observations for traits that have a high impact on fitness in different species, which show that near-zero *h* ^2^ does not necessarily imply the absence of additive genetic variability [30, 55, 56]. In general, the response to selection is G = *ih* ^*2*^σ_P_, where *i* is the selection intensity and σ_P_ is the phenotypic standard deviation [29]. Therefore, if the 5% of the red abalone with strongest immune response are selected from the population as broodstock (*i* = 2.063) we would expect an improvement of the intracellular induction of HSP70, per generation, between 0.6 and 8.1%, and for the extracellular induction of HSP70 between 4.5 and 7.5%, for near-harvest or young adults, respectively. Thus, it can be inferred that despite the low values of *h* ^2^, HSP70 synthesis as an immune response trait in red abalone has the potential to evolve through selection because of the species’ considerable phenotypic variation and the existence of additive genetic variance, especially in young animals [30, 57]. However, the results of the *R* estimates suggest that special environmental variance (*V*_Es_), caused by environmental factors that affect each individual differently throughout its lifespan in a given environment [29], can play a major role influencing the magnitude of the variation in HSP70 production in response to an immunostimulant.

Invertebrates, including molluscs only possess an innate immune system to survive in a pathogen-laden environment [58]. Their immune system relies on circulating cells and a large variety of molecular effectors (humoral components), which are functional proteins present in appropriate amounts, within a critical time window [28]. Invertebrate immune system is not anticipatory, specific, or finely regulated [58], moreover, the prevalence, diversity and rapid evolvability of pathogens suggest that the components of this system may be under constant selection pressure. However, few studies have estimated the *h* ^2^ of humoral components of the immune response, and only three papers have been published on invertebrates, mainly insects [2, 38, 59]. The first two studies measured the activity of gene products in the caterpillar *Spodoptera littoralis*, and a high *h* ^2^ was observed for phenoloxidase activity (PO) (*h* ^*2*^ = 0.65 to 0.69) and antibacterial activity (*h* ^2^ = 0.63) [2, 38]. In addition, moderate *h* ^2^ (0.3–0.4) was observed in the honeybee *Apis mellifera* for the transcription of the gene for abaecin (a key antimicrobial peptide) after challenge with a bacterial pathogen [59]. Transcriptomic studies using microarrays with a large number of genes have shown *h* ^2^ values ranging from 0–1 for the transcription of different genes, with an average of approximately 0.3 [42, 60, 61]. Interestingly, the group of genes associated with the response to unfolded proteins (HSPs and chaperonins) is among those showing the highest *h* ^2^ in humans [61]. In comparison, a low *h* ^2^ of immunoglobulin levels was observed in the bird *Delichon urbica* (*h* ^*2*^ = 0.051) [62]. In this study like in ours, the amount of protein present was evaluated, but gene transcription rates were not assessed; and in both cases, the residual variance was high, which partly explains the phenotypic variance and low *h* ^*2*^ values. These results suggest that the post-transcriptional gene expression processes may contribute non-additive genetic variation to the phenotypic variance, thus resulting in smaller *h* ^2^ values. Although, *h* ^2^ estimates for gene expression of immune genes at transcriptional level are higher, the phenotype of interest for the functional immune response is, of necessity the proteins.

Gene expression analyses have shown that HSP70 is a cytoprotective and anti-apoptotic part of the humoral intracellular response in the immune system of invertebrates [63]. In our study, the abalones subjected to immunostimulation markedly increased (~3-fold) HSP70 levels in haemocytes (intracellular) and serum (extracellular). Furthermore, the induction of this protein in response to an immunostimulant agent (zymosan) was greater in older than in younger adults, suggesting an increase in the immune responsiveness with age. The release of HSP70 into the extracellular medium in response to an immune challenge had not been previously reported in molluscs or other marine invertebrates. However, intracellular and extracellular functions of this protein in the vertebrate immune system have been well studied [4, 15, 16, 64–67]. In addition to its intracellular role, HSP70 is also found extracellularly and bound to the membrane, where it mediates immune function by presenting antigens to cells of the adaptive immune system, also acting as a danger signal and inducing the release of molecules of the innate immune system [4, 17]. This protein could have a similar immune function in vertebrates and invertebrates. Authors have suggested that marine invertebrates share certain characteristics with organisms with acquired immune functions, such as the capacity to enhance specific immune response upon subsequent rechallenge [68–70]. In abalone, high HSP70 levels might result in the extracellular release of HSP70 in serum by haemocytes in response to stimulation by a PAMP. Thus, in addition to its important protein restoring role in response to pathogens, HSP70 could also perform other functions, such as the modulation of the immune response in marine molluscs.

In conclusion, the red abalone responds strongly to challenges with a β-glucan PAMP that increases levels of intracellular and extracellular HSP70. The latter is the first observation of this type in marine molluscs. The *h* ^2^ for intracellular and extracellular HSP70 is low; however, because of the large phenotypic variation and the relatively high *CV*_*A*_, these traits should have a high potential to respond to selection. The question remains as to why a character that appears strongly linked to fitness and is therefore potentially subject to strong directional selection maintains its *V*_A_. The explanation may be that HSP70 is involved in different functions and that optimal expression levels are required for each function, or that divergent selection acts on the HSP70 induction levels depending on the residual genotype or specific environmental conditions during their life span.

## Supporting Information

S1 Fig Western blots showing heat shock protein 70 (HSP70) expressions in abalone haemocytes, serum and gills. Protein extracts (30 μg) from: haemocytes from abalone exposed to heat shock, injected with sterile sea water, injected with zymosan, and control (not injected and not exposed to heat shock) (Hemo-Temp, Hemo-SSW, Hemo-Control, respectively); serum from abalone injected with sterile sea water, injected with zymosan, and control (Serum-SSW, Serum-Zymo, Serum-Control, respectively); and gill tissue from abalone exposed to heat shock (Gill-Temp), were subject to 12% SDS-PAGE followed by Western blot analysis by using a polyclonal mice anti-HSP70 antibody (Group of Immunological Markers on Aquatic Organisms, Catholic University of Valparaiso, Chile), and a goat anti-mouse IgG secondary polyclonal antibody (Thermo Fisher Scientific). Bands were detected using enhanced chemiluminescence (Cyanagen). Molecular mass is shown on the left.
(TIF)
