# Supplementary figures and images for: Potential Response to Selection of HSP70 as a Component of Innate Immunity in the Abalone Haliotis rufescens
Source: PLoS One. 2015 Nov 3;10(11):e0141959. doi: 10.1371/journal.pone.0141959 (PMC4631488; doi:10.1371/journal.pone.0141959)

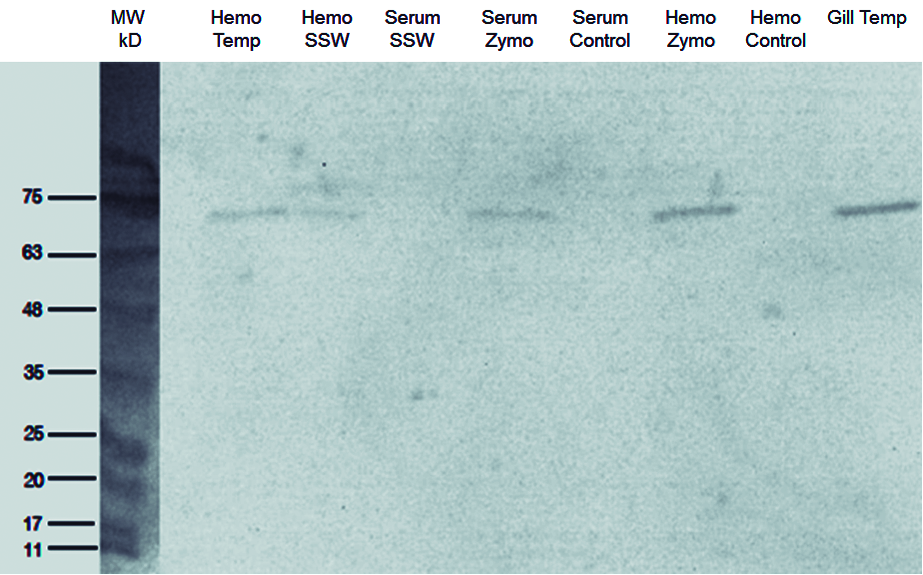

Supplement: S1 Fig — Protein extracts (30 μg) from: haemocytes from abalone exposed to heat shock, injected with sterile sea water, injected with zymosan, and control (not injected and not exposed to heat shock) (Hemo-Temp, Hemo-SSW, Hemo-Control, respectively); serum from abalone injected with sterile sea water, injected with zymosan, and control (Serum-SSW, Serum-Zymo, Serum-Control, respectively); and gill tissue from abalone exposed to heat shock (Gill-Temp), were subject to 12% SDS-PAGE followed by Western blot analysis by using a polyclonal mice anti-HSP70 antibody (Group of Immunological Markers on Aquatic Organisms, Catholic University of Valparaiso, Chile), and a goat anti-mouse IgG secondary polyclonal antibody (Thermo Fisher Scientific). Bands were detected using enhanced chemiluminescence (Cyanagen). Molecular mass is shown on the left. (TIF) [file pone.0141959.s001.tif]
